# Supplementary material for: White matter aberrations and age-related trajectories in patients with schizophrenia and bipolar disorder revealed by diffusion tensor imaging
Source: Sci Rep. 2018 Sep 20;8:14129. doi: 10.1038/s41598-018-32355-9 (PMC6147807; doi:10.1038/s41598-018-32355-9)
Supplement: Supplementary file 1 — Supplemental Information [file 41598_2018_32355_MOESM1_ESM.pdf]

Supplemental Information for

Title: White matter aberrations and age-related trajectories in patients with schizophrenia and bipolar disorder revealed by diffusion tensor imaging

Authors: Siren Tønnesen[1], Tobias Kaufmann[1], Nhat Trung Doan[1], Dag Alnæs[2], Aldo Córdova-Palomera[2], Dennis van der Meer[1], Jaroslav Rokicki[1,3], Torgeir Moberget[2], Tiril P. Gurholt[1], Unn K. Haukvik[1], Torill Ueland[2,3], Trine Vik Lagerberg[2], Ingrid Agartz[1,4], Ole A. Andreassen[1], Lars T. Westlye[2,3*]*

[1]NORMENT, KG Jebsen Centre for Psychosis Research, Institute of Clinical Medicine, University of Oslo, Norway

[2] NORMENT, KG Jebsen Centre for Psychosis Research, Division of Mental Health and Addiction, Oslo University Hospital, Oslo, Norway

[3] Department of Psychology, University of Oslo, Norway

[4] Department of Psychiatric Research, Diakonhjemmet Hospital, Oslo, Norway

** Corresponding authors: Siren Tønnesen (siren.tonnesen@medisin.uio.no) & Lars T. Westlye (l.t.westlye@psykologi.uio.no), postal address: Oslo University Hospital, P.O.Box 4956 Nydalen, 0424 OSLO, Norway, phone: +47 23 02 73 50, Fax: +47 23 02 73 33*

Neuropsychological assessment

Participants completed a neuropsychological test battery, which cover a rage of clinically important neurocognitive domains. Relevant for the present study is current IQ which was measured with the Wechsler Abbreviated Scale of Intelligence (WASI) (1). For details, see (2).

Clinical assessment

Patients went through symptoms assessment (The Positive and Negative Syndrome Scale (PANSS)(3)) and a thorough interview mapping diagnosis, history of disorder, age at onset, hospitalisation, pharmacological treatment and substance use. Psychosocial functioning was assessed with split version of Global Assessment of Functioning Scale (GAF)(4). Trained clinical psychologists and physicians performed clinical, cognitive and diagnostic assessments. For more information, see (5).

Interview at the day of scanning

At the day of scanning, the session started with a short interview and a urine sample to screen for drugs. Patients were asked about recent use of alcohol, drugs and medications, and changes in symptoms since the clinical interview. HCs were asked about recent use of alcohol, drugs and medications, and screened with the Primary Care Evaluation of Mental Disorders (PRIME-MD) (6).

Quality Control

After exclusion, there was a significant group difference for the QC sum score (SZ/BD/HC: -0.13/-0.15/0.08; $F= 3.67$, $p>.026$). Compared to HC and BD, SZ showed higher MAXVOX (2201/1775/1799; $F= 5.08$, $p<.009$), and BD had higher tSNR relative to HC (8.85/7.74/8.88; $F= 3.78$, $p<.024$). Summary stats for the DTI metrics at each QC step are presented in Supplemental Table S5, while Summary Table S6 presents a demographic overview of the participants excluded at each QC step. The excluded participants were visually inspection in order to also manually assess QC. Density plots of DTI metrics within groups for the entire sample (A), for the sample after exclusion of datasets based on quality control and the excluded participants from the quality control are presented in Supplemental Figure S13. In addition, we carried out voxelwise analysis of FA, MD, RD and AD on

the participants that survived the most stringent QC cutoff. The voxel-wise analyses prior to and after QC are presented in Figure 1 and Supplemental Figure S8.

Associations with symptom domains

In line with previous research (7-9) we found no significant associations between WM integrity measures (FA, RD, MD, and AD) and disease severity measured using GAF/PANSS symptom domains. Whereas this lack of sensitivity of DTI metrics to clinically relevant variability does not support a simple dimensional model, it may also suggest the descriptive clinical variables are not readily interpretable or suitable in a dimensional framework targeting mechanisms of disease. Further refinements of both imaging and clinical variables are needed, and further pursuits within a systems neuroscience framework may provide a sensible conceptual and methodological context for improving our understanding of the brain processes underlying complex behaviors and disorders (10).

Diagnostic subgroups

The two major diagnostic groups (SZ and BD) comprise of diagnostic subgroups that might increase the heterogeneity with the groups. Due to the small sample size within some of the subgroups (BD NOS, SA, and SFF) it was not feasible to compare them with the other subgroups within its own spectrum. The results are presented in Supplemental Table S3 and Supplemental Figure S10.

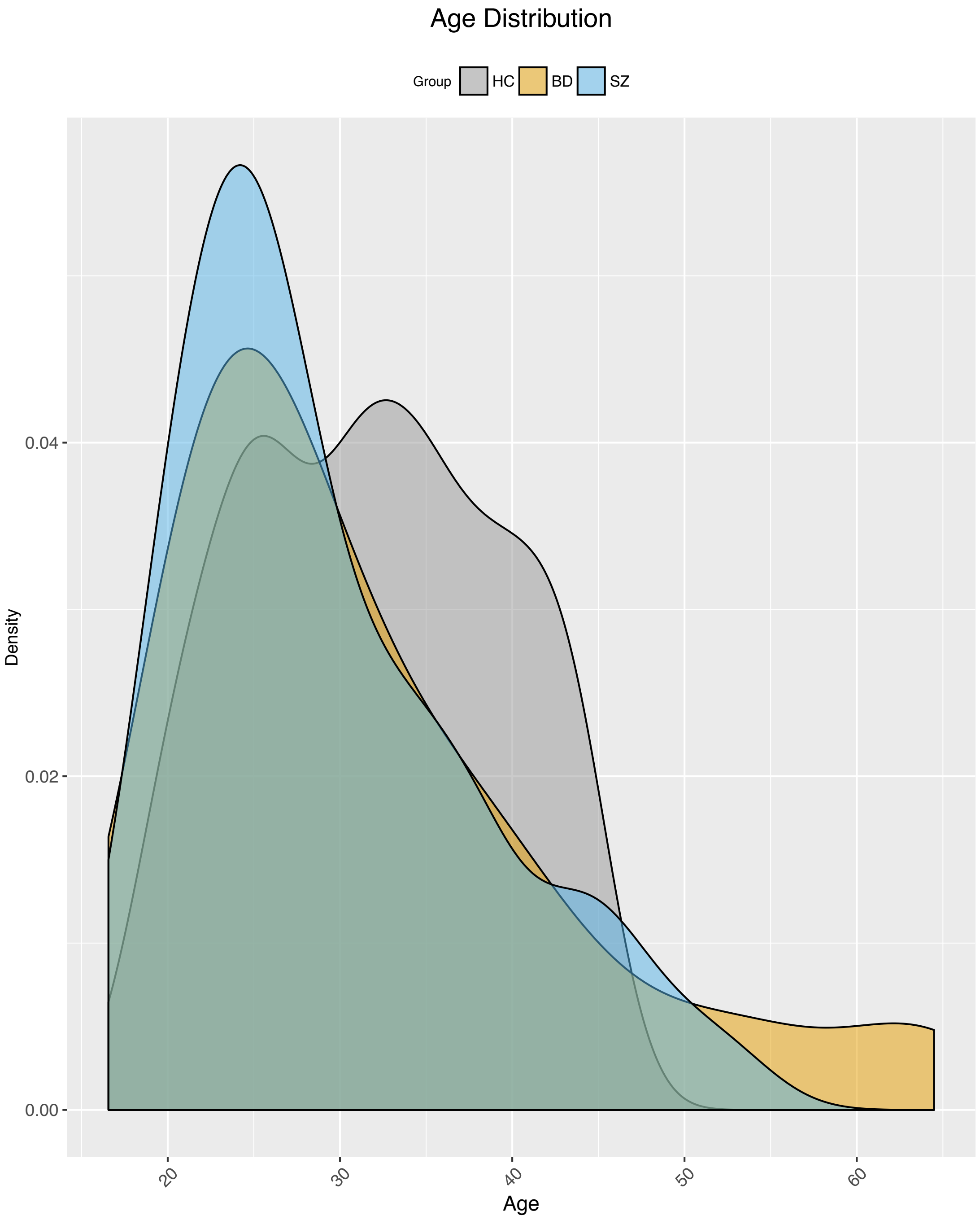

Supplemental Figure S1.
Age distribution within each group, healthy controls (HC), bipolar disorder (BD) and schizophrenia (SZ).

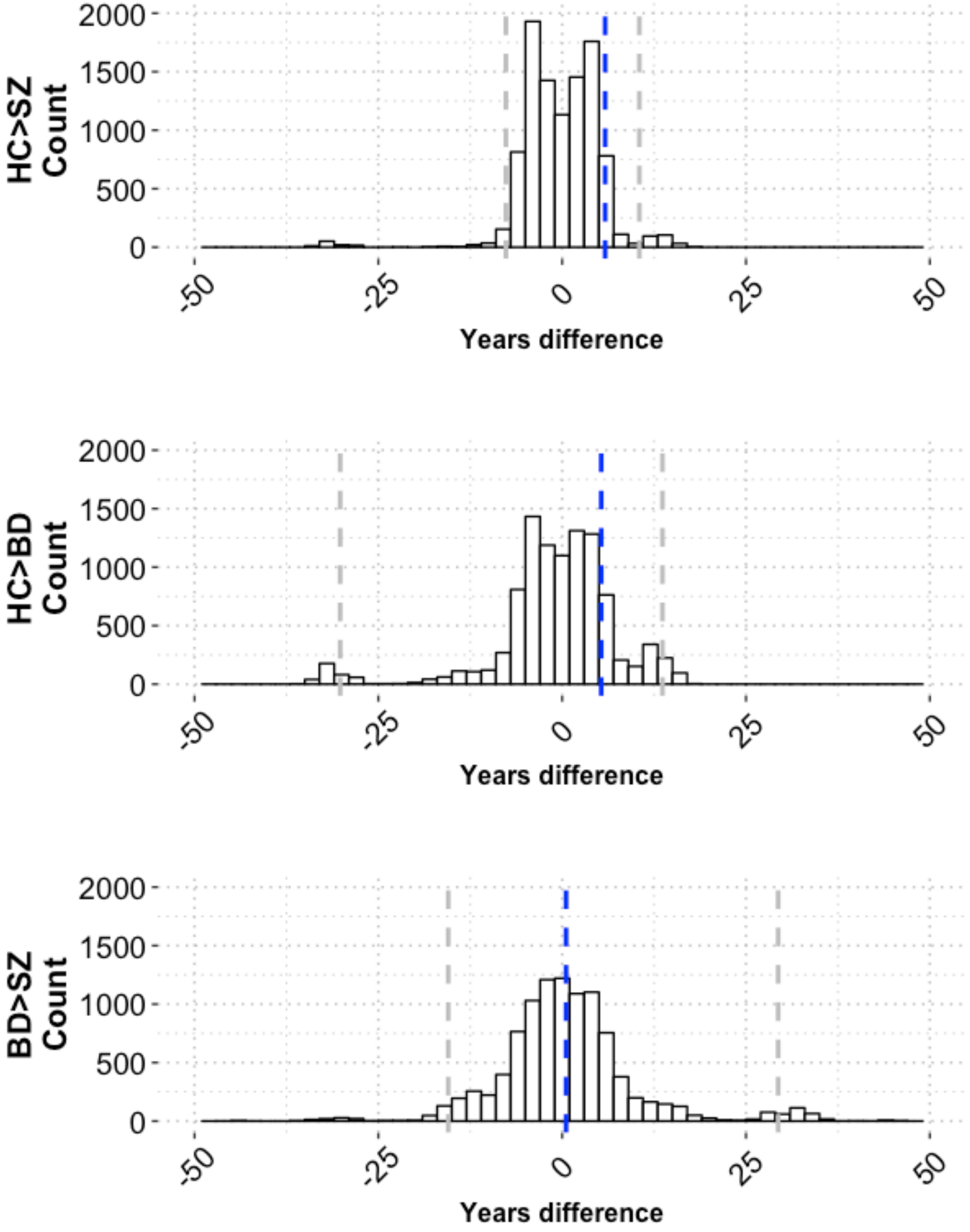

Supplemental Figure S2.
The histogram depicting the empirical null distribution for each contrast generated using permutation testing across 10 000 iterations with the 95% confidence intervals (in grey). The observed age difference between groups is also plotted.

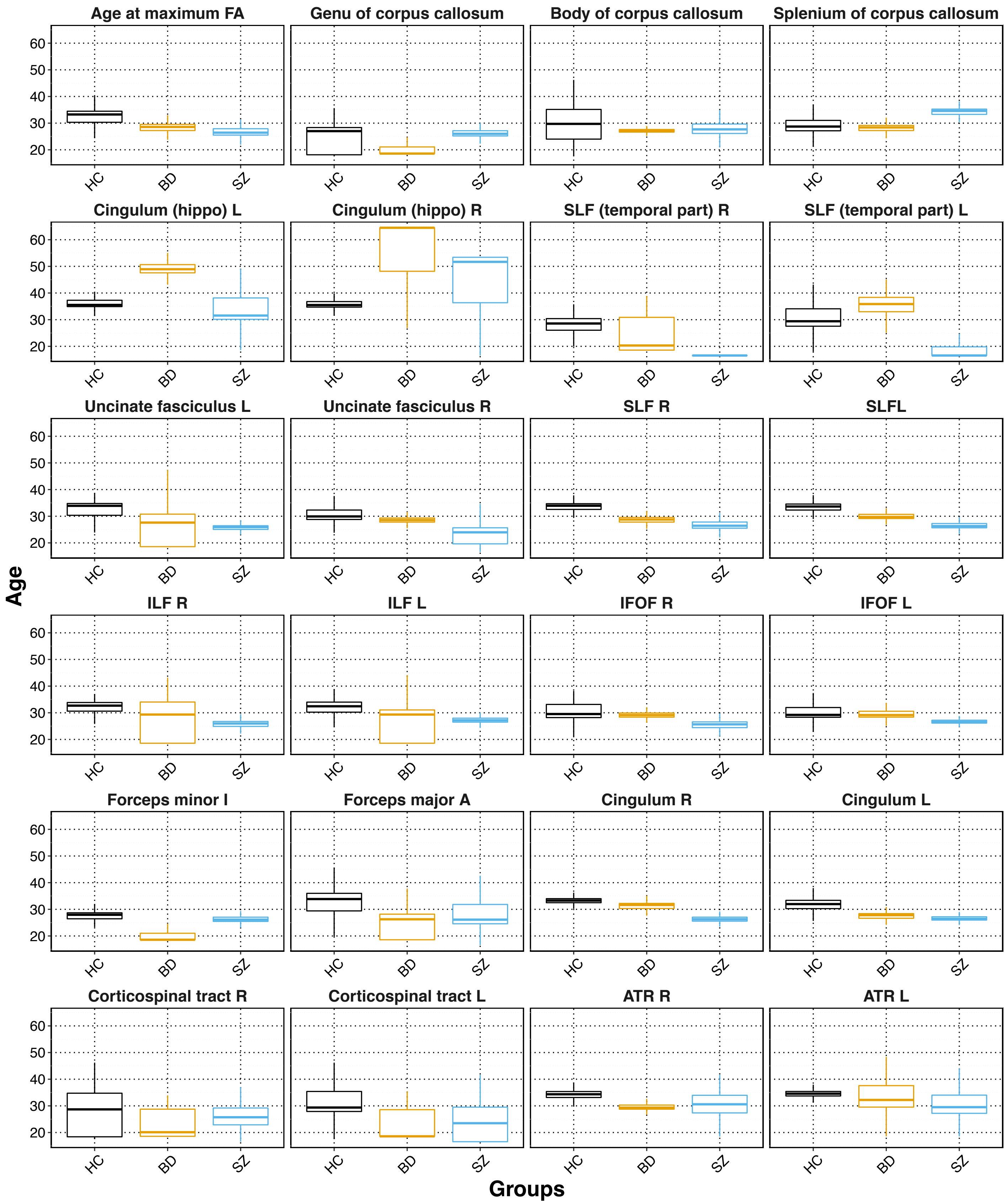

Supplemental Figure S3.

The age when the maximum peak of FA for mean skeleton and 23 regions of interest plotted for each group, healthy controls (HC), bipolar spectrum (BD) and schizophrenia spectrum (SZ). The age estimates were generated from a bootstrap resampling procedure with 10000 iterations. Abbreviations: Superior longitudinal fasciculus (SLF), Inferior longitudinal fasciculus (ILF), Inferior fronto-occipital fasciculus (IFOF), and Anterior thalamic radiation (ATR).

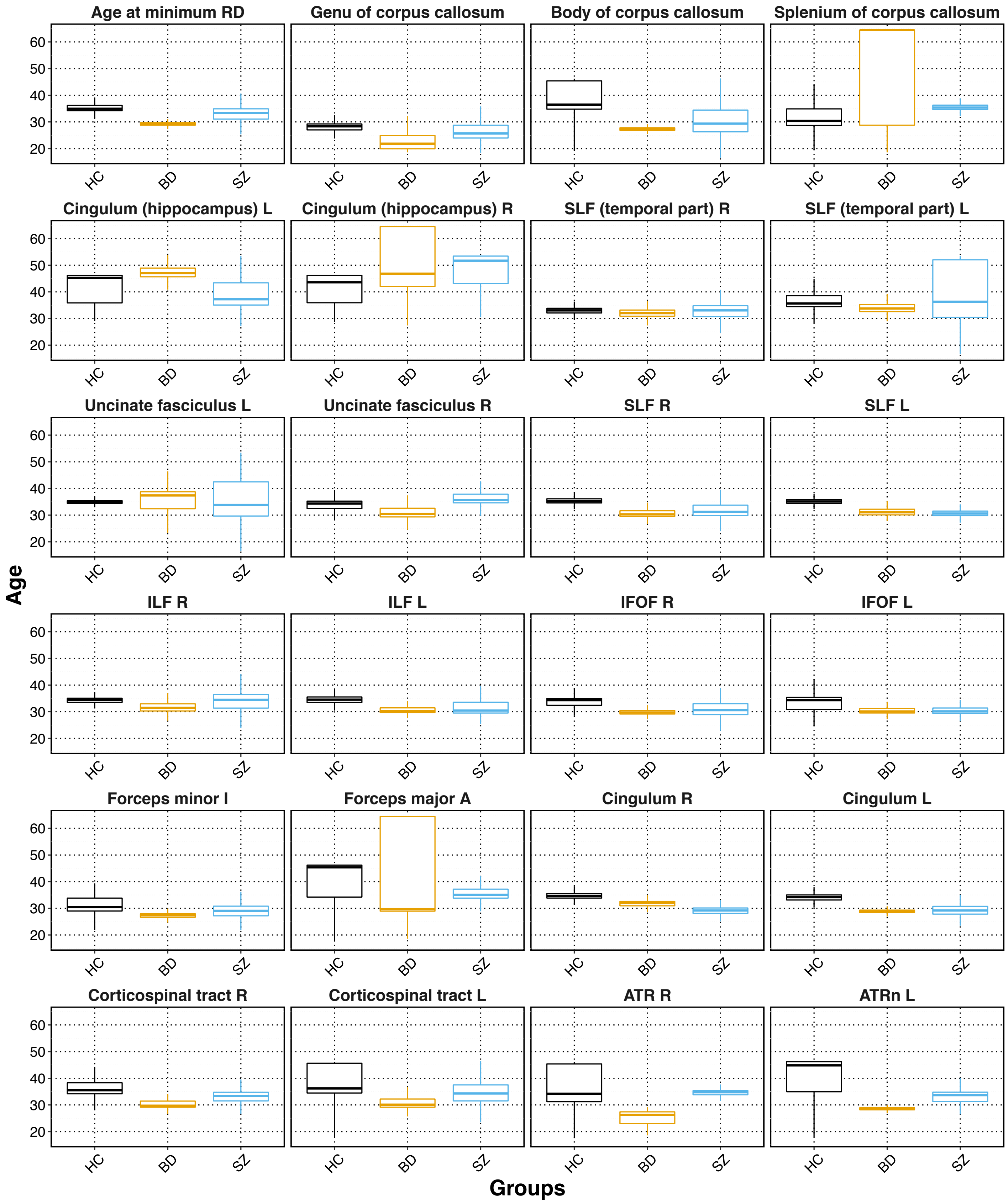

Supplemental Figure S4.

The age when the minimum peak of RD for mean skeleton and 23 regions of interest plotted for each group, healthy controls (HC), bipolar spectrum (BD) and schizophrenia spectrum (SZ). The age estimates were generated from a bootstrap resampling procedure with 10000 iterations. Abbreviations: Superior longitudinal fasciculus (SLF), Inferior longitudinal fasciculus (ILF), Inferior fronto-occipital fasciculus (IFOF), and Anterior thalamic radiation (ATR).

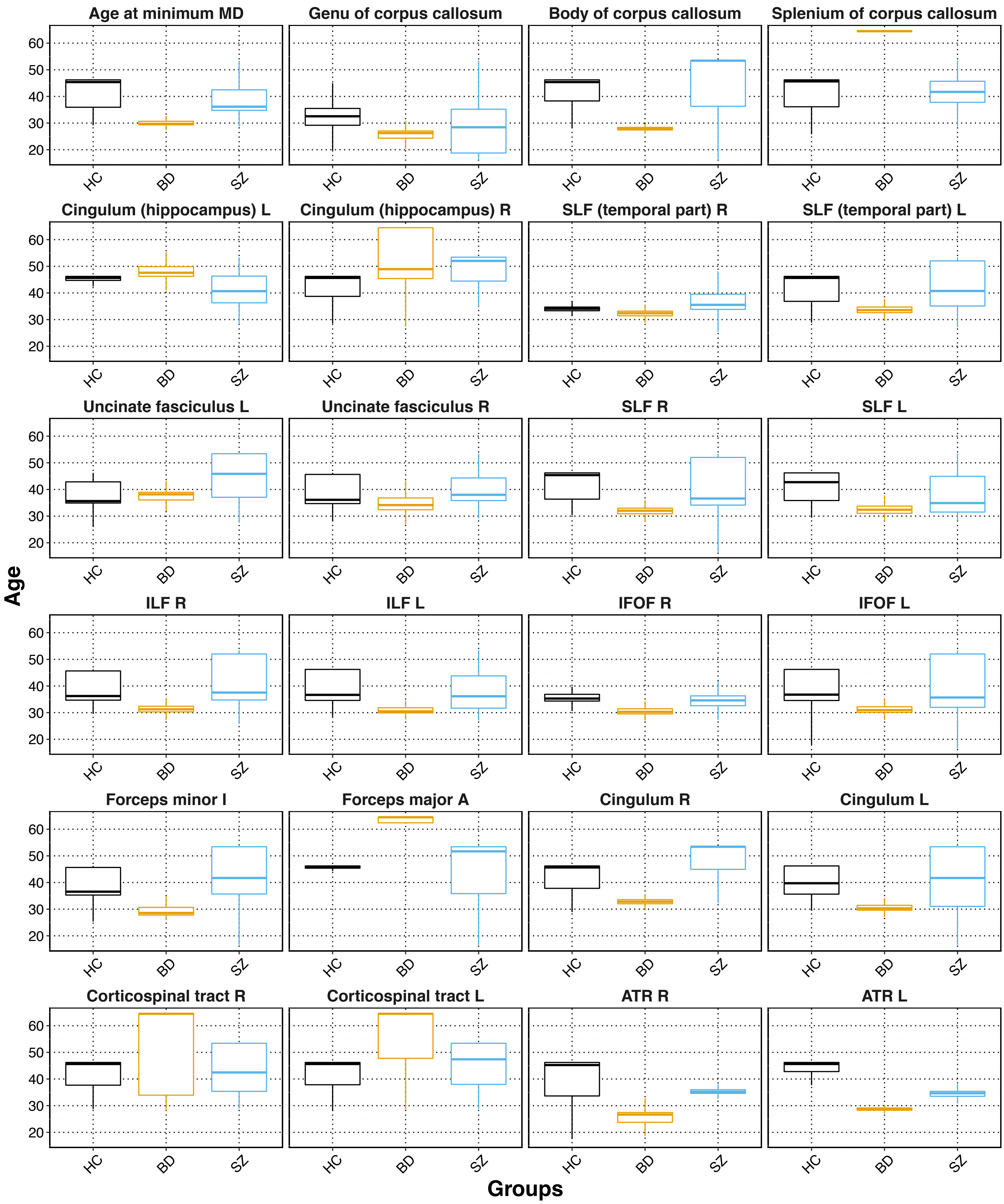

Supplemental Figure S5.

The age when the minimum peak of MD for mean skeleton and 23 regions of interest plotted for each group, healthy controls (HC), bipolar spectrum (BD) and schizophrenia spectrum (SZ). The age estimates were generated from a bootstrap resampling procedure with 10000 iterations. Abbreviations: Superior longitudinal fasciculus (SLF), Inferior longitudinal fasciculus (ILF), Inferior fronto-occipital fasciculus (IFOF), and Anterior thalamic radiation (ATR).

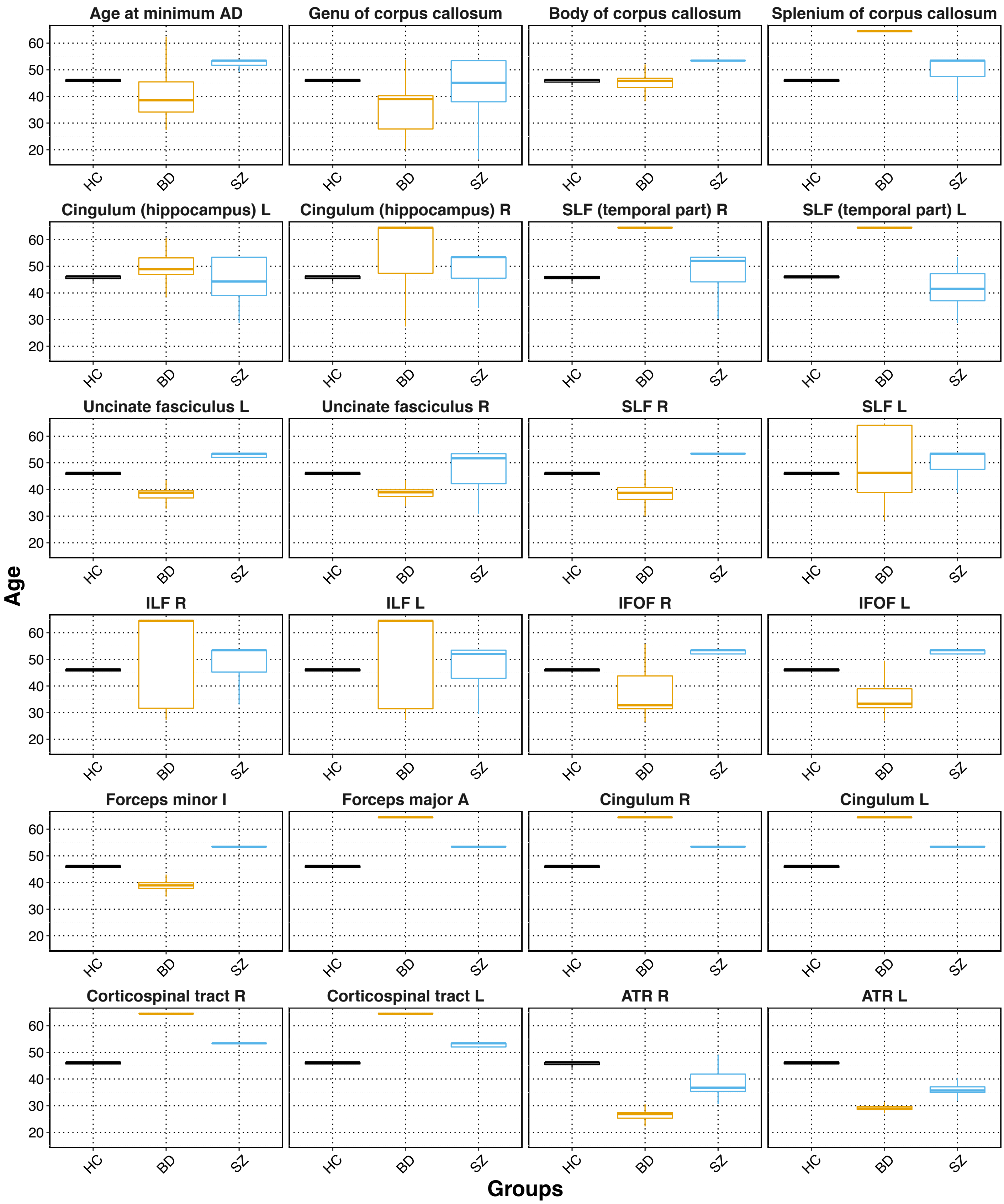

Supplemental Figure S6.

The age when the minimum peak of AD for mean skeleton and 23 regions of interest plotted for each group, healthy controls (HC), bipolar spectrum (BD) and schizophrenia spectrum (SZ). The age estimates were generated from a bootstrap resampling procedure with 10000 iterations. Abbreviations: Superior longitudinal fasciculus (SLF), Inferior longitudinal fasciculus (ILF), Inferior fronto-occipital fasciculus (IFOF), and Anterior thalamic radiation (ATR).

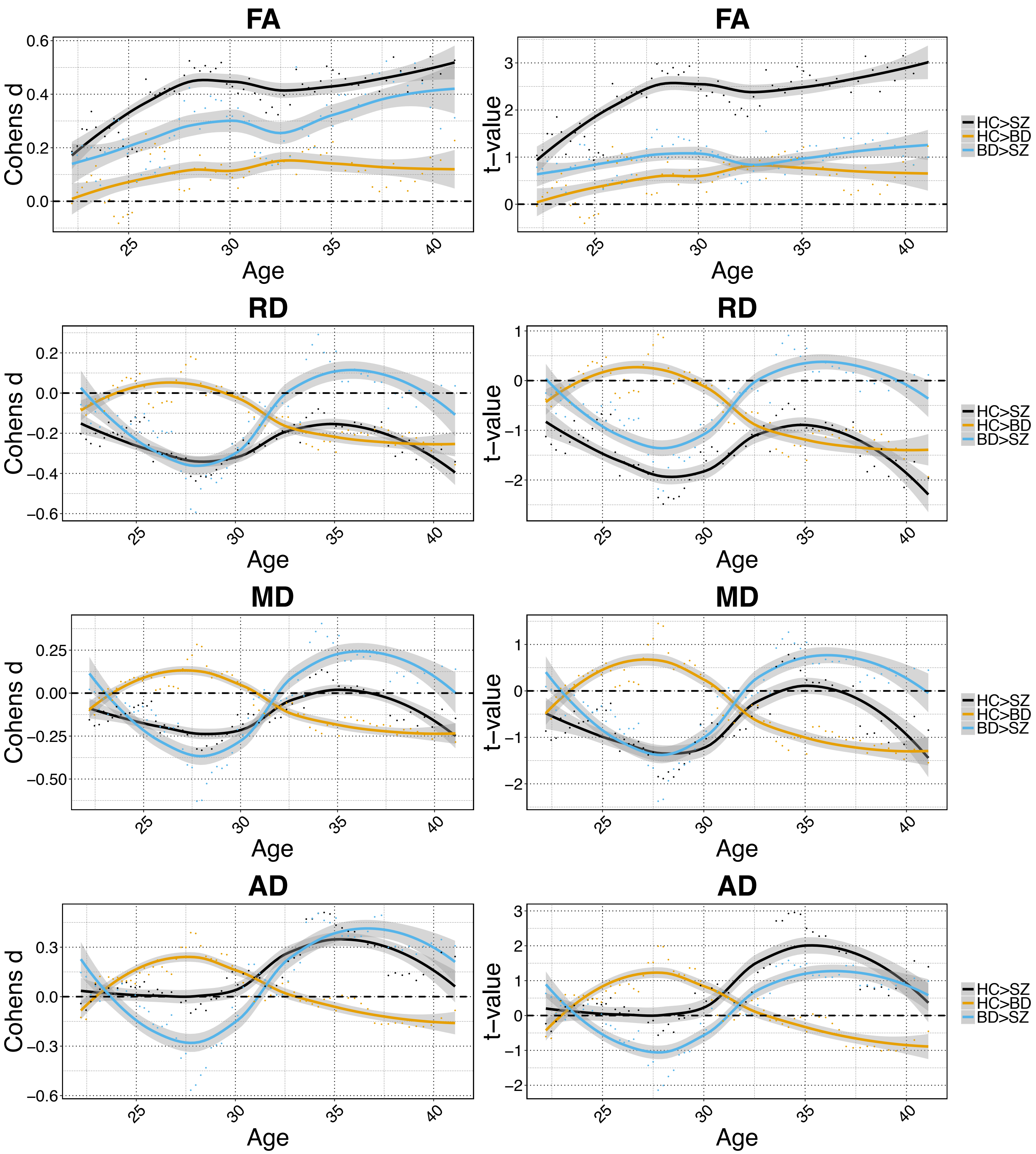

Supplemental Figure S7.

A window of 150 participants in steps of 5 participants were slid along the sorted age span. The resulting Cohens d and t-values resembling pairwise group differences are plotted against the mean age of each sliding group.

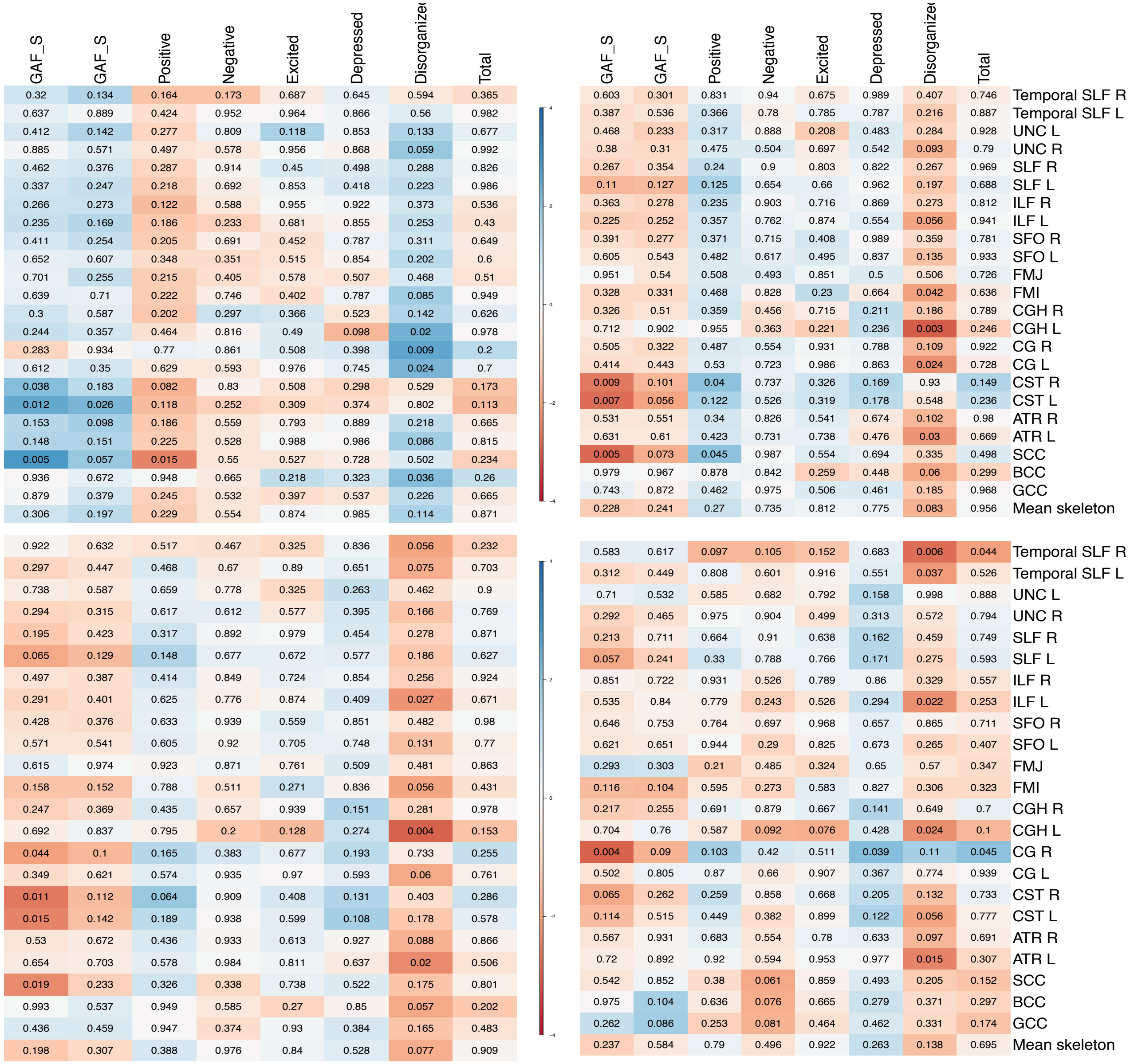

Supplemental Figure S8.

Heat map generated from mean skeleton and ROI analyses reflecting t-values for GAF and PANSS symptom domain scores with the nominal p-values overlaid. No associations were significant when *p* was corrected using false discovery rate (FDR))(11).

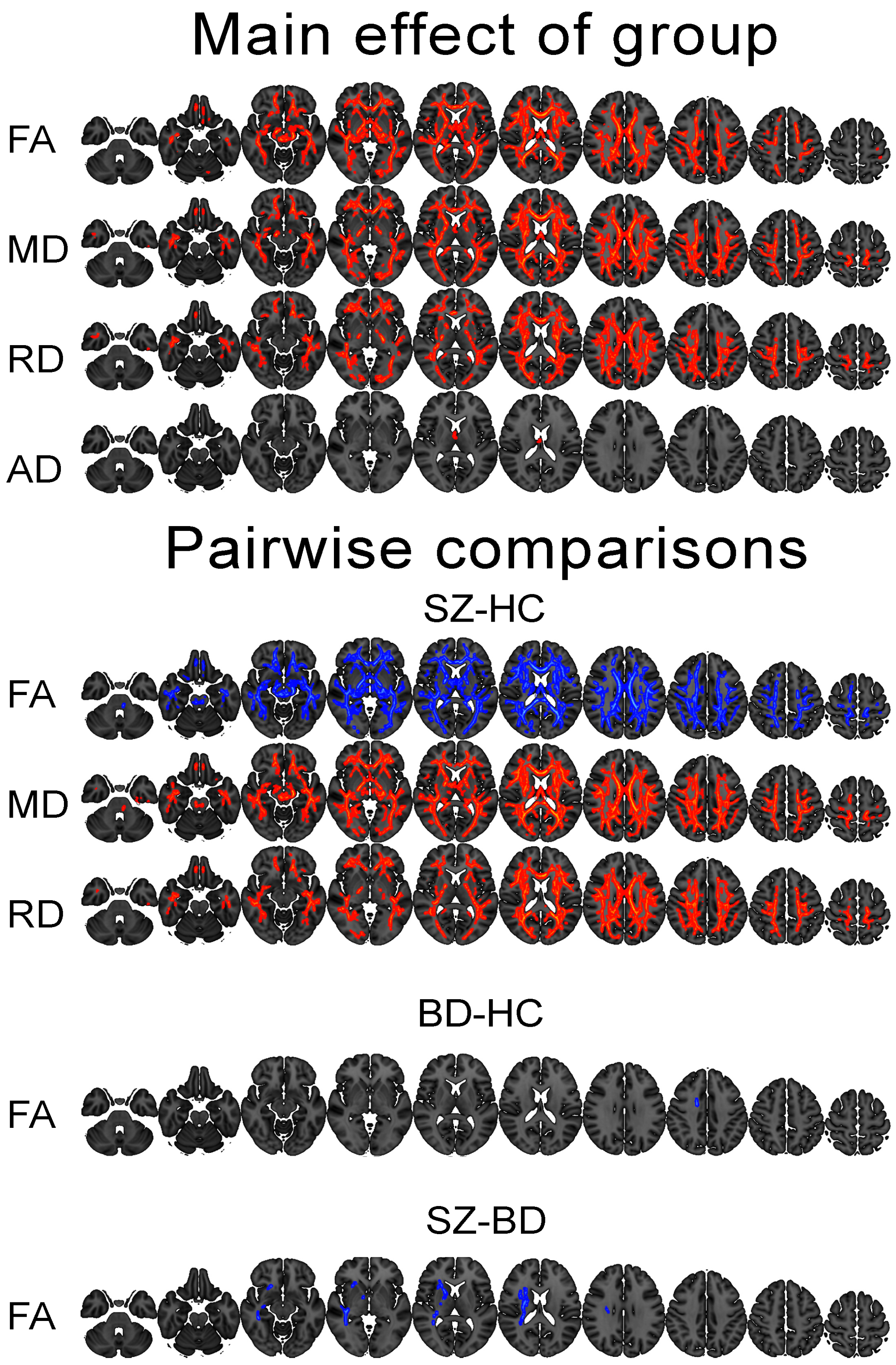

Supplemental Figure S9.
Voxel-wise analyses on participants surviving the most stringent QC step (n=447). Coloured voxels show significantly decreased (blue) and increased (red) DTI-indices in SZ patients relative to HC and BD. Group differences are thresholded at p <.05 (two-tailed) after permutation testing using threshold free cluster enhancement (TFCE). Note that the white matter skeleton has been slightly thickened to aid visualisation.

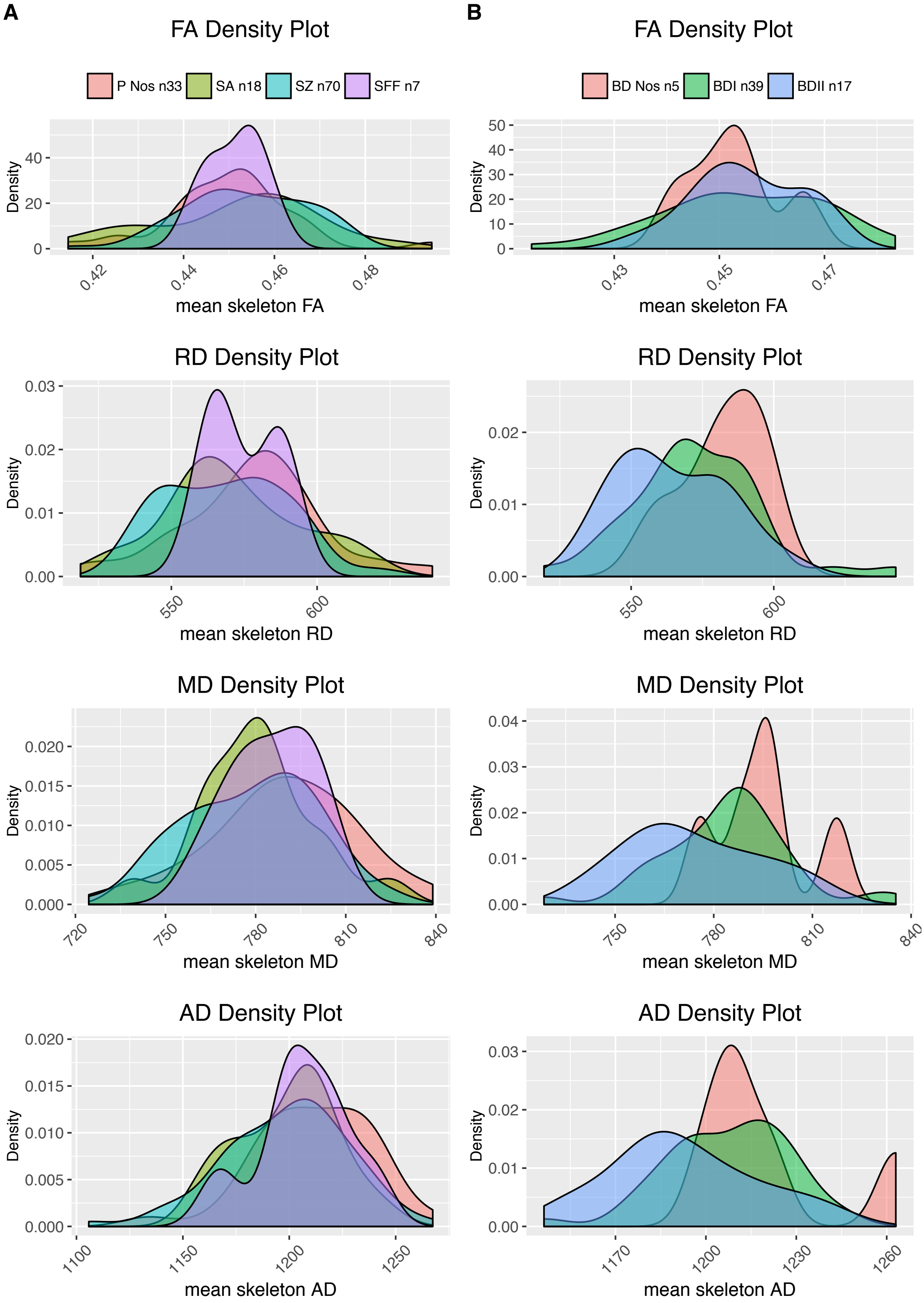

Supplemental Figure S10.

Density plots of mean skeleton DTI metrics for the subgroup of schizophrenia spectrum disorders (A) and bipolar disorders (B). AD, MD and RD are multiplied by 1 000 000 to preserve precision.

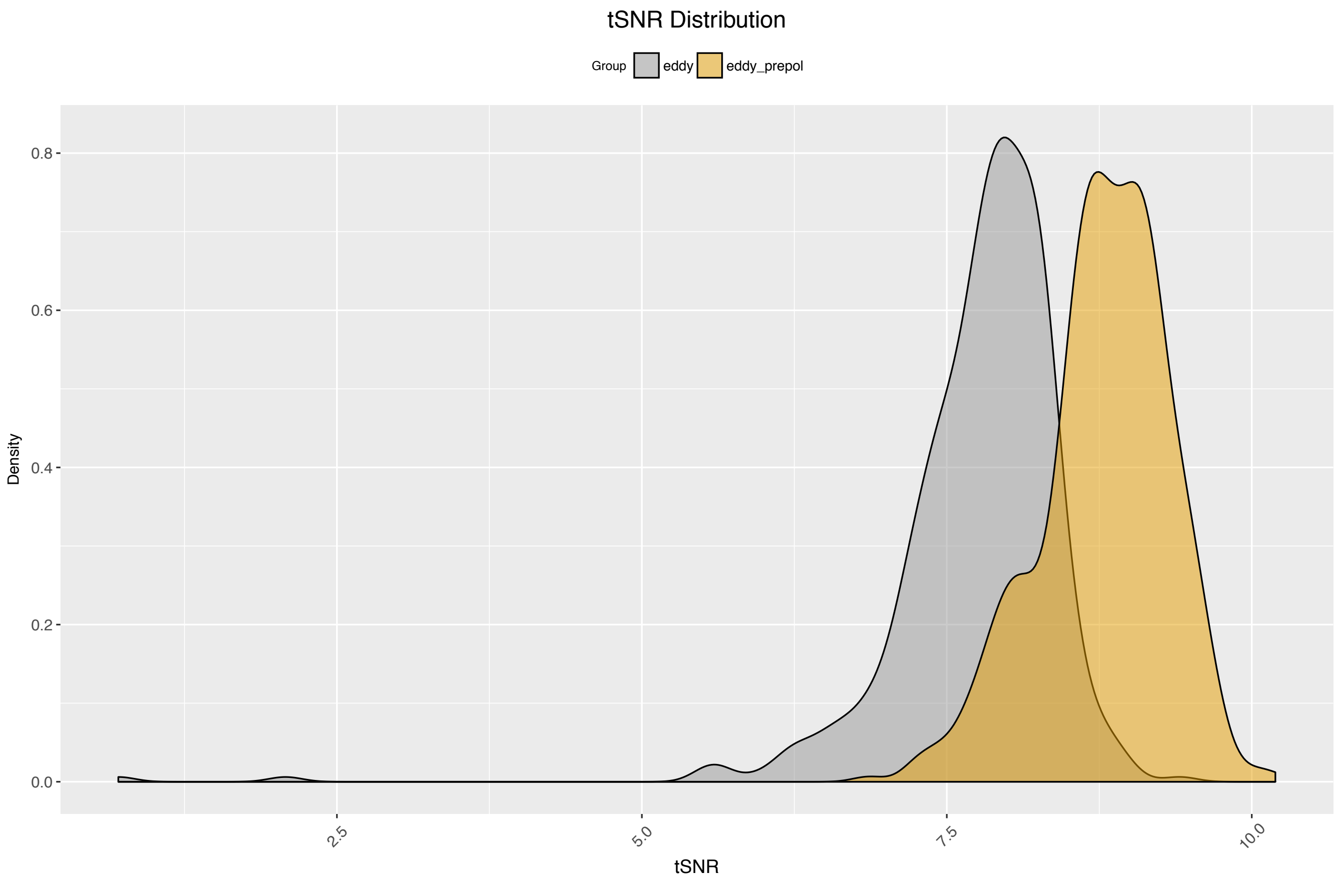

Supplemental Figure S11.
Density plot of the tSNR distribution comparing two versions of Eddy, one with a slice replacement option (eddy_prepol) and one without (eddy).

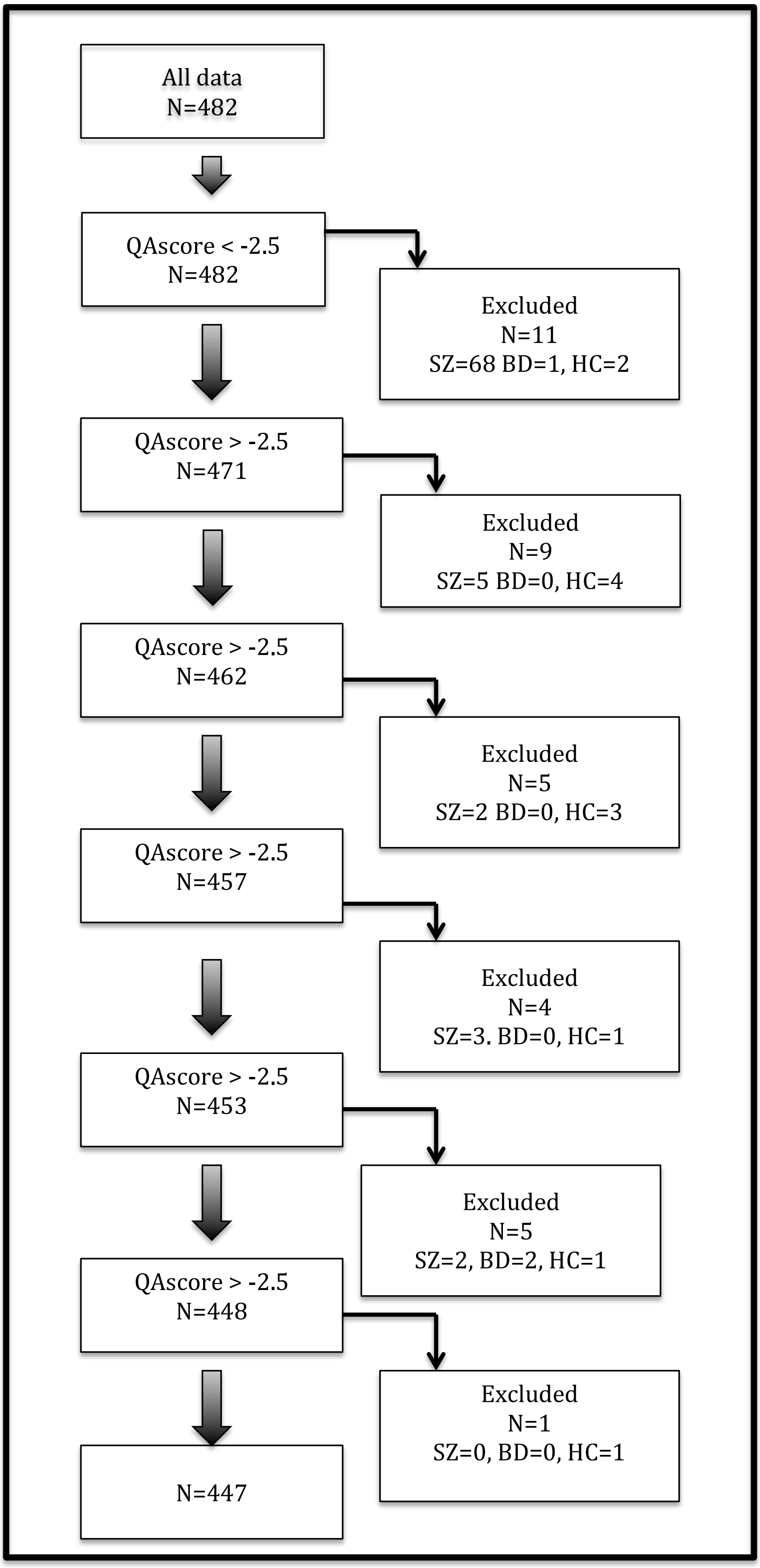

Supplemental Figure S12.
Overview of the quality control procedure, and number of excluded participants at each step. In an iterative fashion, subjects with a QC sum z-score below -2.5 were excluded, and the group statistics were recomputed. This was repeated until no datasets had a z-score below -2.5.

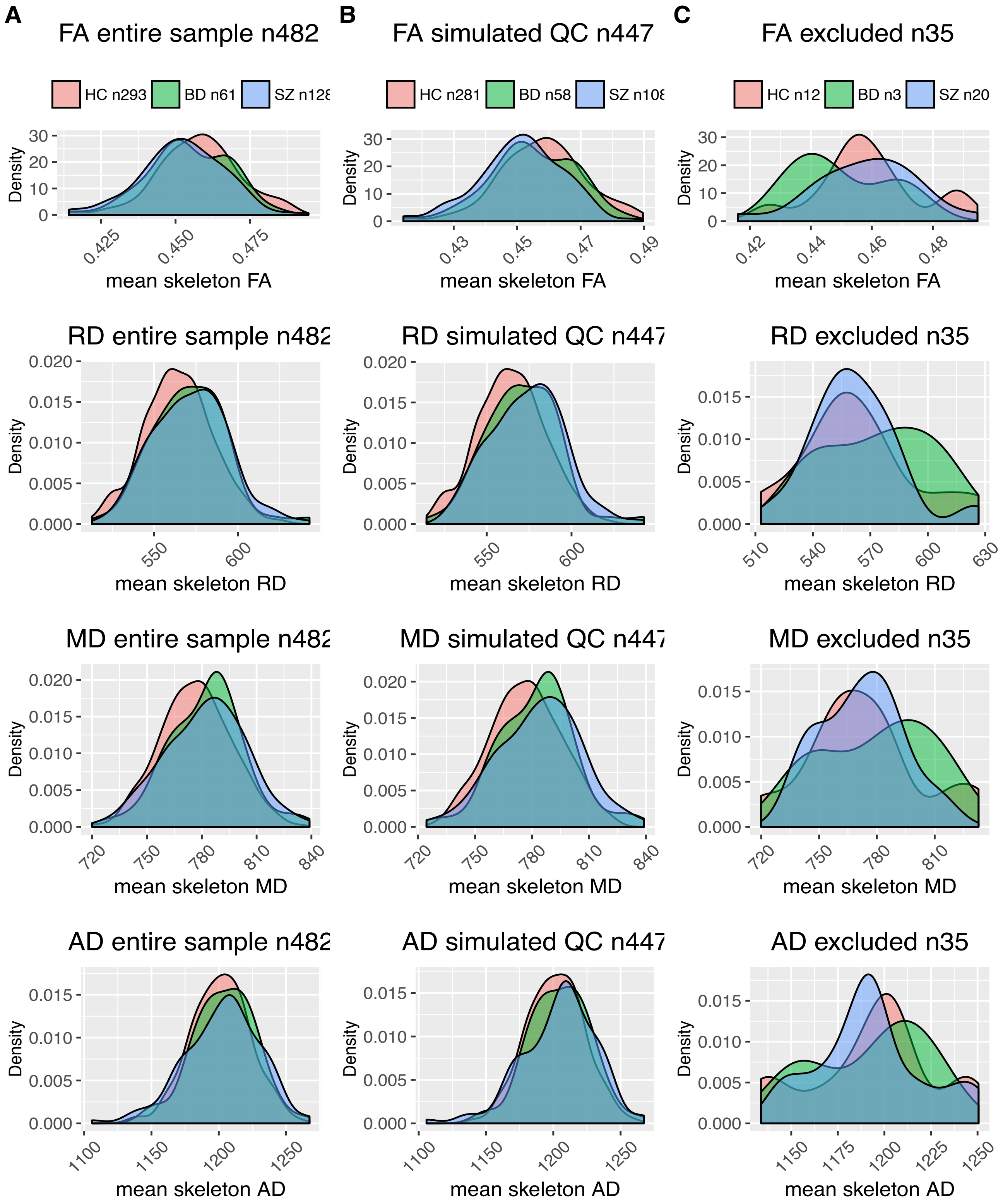

Supplemental Figure S13.

Density plots of DTI metrics within groups for the entire sample (A), for the sample after exclusion of datasets based on quality control (B) and the excluded participants from the quality control (C)

Supplemental Table S1.

Mean skeleton group comparisons within females and males

	SZ	BD	HC	F	p(a)	Pairwise comparison
	Male					
	SZ	BD	HC	F	p(a)	Pairwise comparison
FA	0.455(0.01)	0.455(0.02)	0.461(0.01)	3.36	0.036	HC>SZ
MD	782.01(20.27)	784.60(21.34)	774.92(20.56)	1.22	0.298	
RD	569.52(20.32)	573.50(22.12)	561.97(24.16)	2.21	0.112	
AD	1203.99(26.35)	1206.80(22.80)	1200.81(22.15)	0.71	0.492	
	Female					
FA	0.448(0.01)	0.454(0.02)	0.454(0.01)	3.99	0.020	HC>SZ, BD>SZ
MD	783.60(24.53)	779.85(19.08)	779.27(16.88)	0.49	0.611	
RD	575.29(24.19)	568.85(19.21)	568.08(16.95)	1.57	0.211	
AD	1200.22(30.31)	1201.85(23.21)	1201.65(22.07)	0.70	0.498	

Note. MD,RD,AD multiplied by 1 000 000 to preserve precision. The p-values are not corrected for multiple testing.

Supplemental Table S2.

Association between mean skeleton DTI metrics and GAF and PANSS symptom domain scores across patient groups

	GAF_S	GAF_F	Positive	Negative	Depressed	Disorganized	Excited
	t(p)	t(p)	t(p)	t(p)	t(p)	t(p)	t(p)
FA	1.03 (.306)	1.29(.197)	-1.21(.229)	-0.59(.554)	0.02(.985)	1.59(.114)	0.16(.874)
MD	-1.29(.198)	-1.02(.308)	0.97(.388)	-0.03(.976)	0.63(.528)	-1.78(.077)	0.20(.840)
RD	-1.21(.228)	-1.18(.241)	1.11(.270)	0.34(.735)	0.28(.775)	-1.74(0.083)	0.24(.812)
AD	-1.19(.238)	-0.55(.584)	0.27(.790)	-0.68(.50)	1.1(.263)	-1.50(.138)	0.10(.922)

Note. PANSS is an abbreviation for The Positive and Negative Syndrome Scale, while GAF is Global Assessment of Functioning Scale. GAF_F refers to the functioning subscale while GAF_S is the symptom subscale. The p-values are not corrected for multiple testing

Supplemental Table S3.

Mean skeleton DTI metrics for each subgroup for each of the two diagnostic groups (SZ and BD).

	**Schizophrenia Spectrum Disorders (SZ)**							Pairwise comparison
	SZ# (n=70) Mean(SD)	SA (n=18) Mean(SD)	SFF (n=7) Mean(SD)	PNOS# (n=33) Mean(SD)	HC# (n=293) Mean(SD)	F	p(a)	
FA	0.453(0.01)	0.452(0.02)	0.451(0.01)	0.450(0.02)	0.458(0.01)	5.80	0.003	HC>SZ, HC>PNOS
MD	778.69(21.73)	780.34(19.47)	785.34(14.10)	789.40(24.11)	776.71(19.22)	4.59	0.011	PNOS>SZ
RD	568.82(20.91)	570.88(23.23)	574.70(11.88)	578.09(24.68)	564.49(20.34)	4.79	0.010	HC<PNOS
AD	1198.42(29.25)	1199.26(23.25)	1206.62(22.62)	1212.02(28.03)	1201.16(22.08)	6.60	0.002	PNOS>HC>SZ
	**Bipolar Spectrum Disorders (BD)**							
	BDI# (n=39) Mean(SD)	BDII# (n=17) Mean(SD)	BDNOS (n=5) Mean(SD)		HC# (n=293) Mean(SD)	F	p(a)	
FA	0.455(0.02)	0.455(0.01)	0.453(0.01)		0.458(0.01)	0.90	0.408	
MD	784.08(19.61)	773.85(20.49)	794.86(15.17)		776.71(19.22)	3.38	0.035	HC>BDII, BDII<BDI
RD	572.58(22.77)	564.36(19.50)	582.03(14.77)		564.49(20.34)	2.79	0.062	
AD	1201.07(20.39)	1192.83(24.32)	1220.54(24.99)		1201.16(22.08)	3.39	0.035	HC<BDII, BDI>BDII

Note. MD,RD,AD multiplied by 1 000 000 to preserve precision. The p-values are not corrected for multiple testing. #Due to small subgroup sizes, subgroup comparisons were only conducted for the HC/SZ/PNOS and the HC/BDI/BDII contrasts. The subgroups within the schizophrenia spectrum (SZ) were: schizophrenia (SZ), schizoaffective (SA), schizophreniform (SFF) and psychosis not otherwise specificed (SFF). The subgroups within the bipolar spectrum were: bipolar 1 disorder (BDI), bipolar 2 disorder (BDII) and bipolar disorder not otherwise specificed (BDNOS).

Supplemental Table S4.

Mean skeleton DTI metrics within groups with age restriction[a]

	SZ Mean(SD)	BD Mean(SD)	HC Mean(SD)	F	$p^{(a)}$	Pairwise comparison
FA	0.452(0.01)	0.455(0.01)	0.458(0.01)	6.33	<0.001	HC>SZ
MD	782.04(22.01)	781.08(18.51)	776.71(19.22)	2.81	0.448	
RD	571.82(22.03)	569.53(19.23)	564.49(20.34)	2.82	0.061	
AD	1202.49(27.94)	1204.18(22.47)	1201.16(22.08)	0.61	0.542	

Note. MD,RD,AD multiplied by 1 000 000 to preserve precision. The p-values are not corrected for multiple testing.

[a] – analyses were done on participants aged 55 years and younger

Supplemental Table S5.

Breakdown of Quality Control

N	SZ	BD	HC	F	$p^{(a)}$	Pairwise comparison
**QC score**						
n482	-0.34	-0.04	0.15	10.42	0.003	HC>SZ, BD>SZ
n471	-0.23	-0.07	0.11	4.94	0.007	HC>SZ
n462	-0.18	-0.15	0.10	4.56	0.011	HC>SZ
n457	-0.18	-0.17	0.11	5.30	0.005	HC>SZ, HC>BD
n453	-0.14	-0.20	0.10	4.61	0.010	HC>SZ, HC>BD
n448	-0.12	-0.15	0.08	3.45	0.033	HC>SZ, HC>BD
n447	-0.13	-0.15	0.08	3.67	0.026	HC>SZ, HC>BD
**tSNR**						
n482	8.72	8.70	8.84	6.61	0.002	HC>SZ, HC>BD
n471	8.76	8.72	8.85	3.22	0.041	HC>SZ, HC>BD
n462	8.80	8.72	8.86	3.24	0.040	HC>BD
n457	8.81	8.72	8.87	4.30	0.014	HC>BD
n453	8.84	8.72	8.88	4.40	0.013	HC>BD
n448	8.85	8.74	8.88	3.83	0.022	HC>BD
n447	8.85	8.74	8.88	3.78	0.024	HC>BD
**Maxvox**						
n482	3326.15	2068.56	1983.36	15.26	<0.001	HC<SZ, HC<BD
n471	2586.25	1872.17	1937.35	7.70	<0.001	HC<SZ, BD<SZ
n462	2391.72	1872.17	1853.40	6.89	<0.001	HC<SZ, BD<SZ
n457	2337.87	1872.17	1839.40	6.20	0.002	HC<SZ, BD<SZ
n453	2279.13	1872.17	1841.45	4.80	0.009	HC<SZ, BD<SZ
n448	2201.19	1775.48	1821.58	4.36	0.013	HC<SZ, BD<SZ
n447	2201.19	1775.48	1799.95	5.08	0.007	HC<SZ, BD<SZ

Note. Subjects with a QC sum z-score below -2.5 were excluded, and the group statistics were recomputed. This was repeated until no datasets had a z-score below -2.5. In total there were six rounds of exclusion before no datasets had a z-score below -2.5. n482 is the complete dataset, n471 is the first round of exclusion and the subsequent reduction of number of participants (n471, n462, n457, n453, n448, n447) indicates the successive rounds of exclusions.

Supplemental Table S6.

Demographical overview of excluded patients and controls

	SZ	BD	HC
**All excluded (n=35)**			
N	20	3	12
Age	28.61(8.9)	53.39(17.4)	30.94(9.9)
Sex, *n*, (% male)	15(75%)	1(33%)	9(75%)
**N471 (n=11) First round of exclusion**			
N	8	1	2
Age	27.27(8.5)	64.48(NA)	37.16(8.3)
Sex, *n*, (% male)	5(63%)	0(0%)	2(100%)
**N462 (n=9) Second round of exclusion**			
N	5	0	4
Age	29.21(8.6)	NA	22.49(5.8)
Sex, *n*, (% male)	5(100%)	NA	4(100%)
**N457 (n=5) Third round of exclusion**			
N	2	0	3
Age	23.30(1.6)	NA	26.11(4.1)
Sex, *n*, (% male)	2(100%)	NA	1(33%)
**N453 (n=4) Fourth round of exclusion**			
N	3	0	1
Age	33.86(15.6)	NA	42.43(NA)
Sex, *n*, (% male)	2(66%)	NA	1(100%)
**N448 (n=5) Fifth round of exclusion**			
N	2	2	1
Age	29.91(5.9)	48.85(20.6)	43.14(NA)
Sex, *n*, (% male)	1(50%)	1(50%)	0(0%)
**N447 (n=1) Sixth round of exclusion**			
N	0	0	1
Age	NA	NA	43.14(NA)
Sex, *n*, (% male)	NA	NA	1(100%)

Note. Subjects with a QC sum z-score below -2.5 were excluded, and the group statistics were recomputed. This was repeated until
no datasets had a z-score below -2.5

References

1. Wechsler D (2007): *Wechsler Abbreviated Scale of Intelligence (WASI). Norwegian Manual Supplement* Stockholm, Sweden: Pearson Assessment.
2. Simonsen C, Sundet K, Vaskinn A, Birkenaes AB, Engh JA, Faerden A, et al. (2011): Neurocognitive dysfunction in bipolar and schizophrenia spectrum disorders depends on history of psychosis rather than diagnostic group. *Schizophrenia bulletin*. 37:73-83.
3. Kay SR, Fiszbein A, Opler LA (1987): The positive and negative syndrome scale (PANSS) for schizophrenia. *Schizophrenia bulletin*. 13:261-276.
4. Pedersen G, Hagtvet KA, Karterud S (2007): Generalizability studies of the Global Assessment of Functioning-Split version. *Comprehensive psychiatry*. 48:88-94.
5. Vaskinn A, Sundet K, Simonsen C, Hellvin T, Melle I, Andreassen OA (2011): Sex differences in neuropsychological performance and social functioning in schizophrenia and bipolar disorder. *Neuropsychology*. 25:499-510.
6. Spitzer RL, Williams JB, Kroenke K, Linzer M, deGruy FV, 3rd, Hahn SR, et al. (1994): Utility of a new procedure for diagnosing mental disorders in primary care. The PRIME-MD 1000 study. *Jama*. 272:1749-1756.
7. Roalf DR, Gur RE, Verma R, Parker WA, Quarmley M, Ruparel K, et al. (2015): White matter microstructure in schizophrenia: associations to neurocognition and clinical symptomatology. *Schizophrenia research*. 161:42-49.
8. Kochunov P, Ganjgahi H, Winkler A, Kelly S, Shukla DK, Du X, et al. (2016): Heterochronicity of white matter development and aging explains regional patient control differences in schizophrenia. *Human brain mapping*
9. McIntosh AM, Munoz Maniega S, Lymer GK, McKirdy J, Hall J, Sussmann JE, et al. (2008): White matter tractography in bipolar disorder and schizophrenia. *Biological psychiatry*. 64:1088-1092.
10. Frangou S (2014): A Systems Neuroscience Perspective of Schizophrenia and Bipolar Disorder. *Schizophrenia bulletin*.
11. Benjamini Y, Hochberg Y (1995): Controlling the False Discovery Rate: A Practical and Powerful Approach to Multiple Testing. . *Journal of the Royal Statistical Society Series B (Methodological),* . 57:289-300.
